# HbA1c measurement using capillary blood in epidemiological studies: a scoping review of technologies and approaches

**DOI:** 10.1186/s12902-026-02410-y

**Published:** 2026-07-11

**Authors:** Bruna Suellen Breternitz, Raquel Queiroz de Araújo, Edna Terezinha Rother, Alice Barone de Andrade, Paula Carvalho de Freitas, Luiz Menezes-Júnior, Oscar Geovanni Enriquez-Martinez, Leticia de Oliveira Cardoso, Michele Lacerda Ferrer, Etienne Duim

**Affiliations:** 1 Hospital Israelita Albert Einstein, São Paulo, SP Brazil; 2 Ministério da Saúde (MS), Secretaria de Vigilância em Saúde e Ambiente (SVSA), Departamento de Análise Epidemiológica e Vigilância de Doenças Não Transmissíveis (DAENT), Brasília, Brazil; 3 Departamento de Atenção Primária à Saúde - Faculdade de Ciências Médicas Santa Casa de São Paulo, São Paulo, Brazil

**Keywords:** Glycated hemoglobin (HbA1c), Point of care testing (PoCT), Dried blood spot (DBS), Health surveys, Global health equity

## Abstract

**Objective:**

To map and describe the capillary blood–based technologies employed to measure glycated hemoglobin (HbA1c) in epidemiological studies, focusing on their accuracy, applicability, feasibility, and implementation barriers.

**Introduction:**

The development of technologies for diabetes screening and monitoring has expanded, particularly in decentralized and epidemiological contexts.

**Methods:**

A scoping review was conducted in accordance with PRISMA-ScR guidelines. A comprehensive literature search was performed in Embase, Scopus, Web of Science, LILACS, and CINAHL between 18 and 24 February 2025. The search was conducted without restrictions on language, publication year, or geographic location, retrieving studies indexed up to 31 January 2025. We included primary epidemiological studies involving adults (≥ 18 years) that assessed HbA1c measurement using capillary blood technologies or approaches in field or population-based settings. Studies exclusively evaluating capillary glucometers, continuous glucose monitoring, or oral glucose tolerance tests were excluded. Two reviewers independently screened records and assessed full-text eligibility against pre-specified criteria registered on the Open Science Framework (OSF). Data were extracted and synthesized descriptively based on outcomes reported by the original study authors and categorized in regard to the aim.

**Results:**

Of 2,238 records screened, 106 studies were included. Fifteen distinct analytical technologies for HbA1c measurement using capillary blood were identified, with Point-of-Care Testing (PoCT) devices representing the predominant category. Among studies reporting diagnostic accuracy metrics, sensitivity ranged from 17.4% to 100% and specificity from 43.5% to 99.1%. PoCT devices required 2–10 µL of capillary blood and produced results within minutes. Applicability findings described portability, limited training requirements, and use in decentralized settings. Dried Blood Spot (DBS) approaches facilitated remote collection and mailing to reference laboratories. Reported challenges included variability in analytical performance, limited validation in Low- and Middle-Income Countries (LMICs), and operational constraints related to cost, supply logistics, and integration into health systems. Overall, 77% of included studies were conducted in High-Income Countries (HICs).

**Conclusions:**

Capillary blood–based technologies for HbA1c measurement have been applied across diverse epidemiological contexts, with reported analytical and operational characteristics consistent with decentralized use. Nevertheless, the evidence remains geographically concentrated and heterogeneous, indicating gaps in research from LMICs.

**Supplementary Information:**

The online version contains supplementary material available at https://doi.org/10.1186/s12902-026-02410-y.

## Introduction

Diabetes Mellitus (DM) has emerged as one of the most pressing public health emergencies in contemporary societies. Global estimates suggest that over 1.31 billion people may be living with the disease by 2050 [1], with particularly rapid growth observed in Low- and Middle-Income Countries (LMICs), where persistent barriers to diagnosis and treatment remain [2, 3]. In the Americas, disability-adjusted life years (DALYs) attributable to DM have increased by approximately 17% over the past two decades [4]. Given these trends, curbing the progression of DM and its chronic complications has become a priority for health systems worldwide.

Early detection is a strategy to reduce disease progression and prevent DM related complications [3]. Traditional screening and diagnostic methods, while clinically valuable, involve logistical and financial constraints that hinder their applicability in large-scale surveys, particularly in remote or resource-limited contexts [5]. As a result, vulnerable populations remain underdiagnosed, and health disparities continue.

Population-based surveys are essential tools for monitoring noncommunicable chronic diseases (NCDs), guiding resource allocation, and informing health policies. In Brazil, national household surveys such as the National Health Survey (Pesquisa Nacional de Saúde [PNS]) and the National Household Sample Survey (Pesquisa Nacional por Amostra de Domicílios [PNAD]) have shed light on the burden of DM and primary healthcare coverage [6, 7]. Nonetheless, reliance on self-reported data, need for specialized laboratories, and high operational costs limit the precision and reach of these initiatives.

HbA1c is a biomarker that reflects mean glycemic control over the previous two to three months. It demonstrates greater pre-analytical stability and does not require prior fasting [8, 9]. It provides an estimate of average plasma glucose using a single non-fasting sample, thereby eliminating the need for timed collections and special patient preparation. HbA1c-defined intermediate hyperglycemia is based on American Diabetes Association (ADA) criteria, with prediabetes defined as 5.7–6.4% and diabetes diagnosis as HbA1c ≥ 6.5% [8]. Its practicality and analytical robustness support its role as a gold-standard marker for glycemic control and a valuable option for screening [9].

Capillary blood-based technologies and Point-of-Care Testing (PoCT) devices have emerged as promising alternatives, offering a more accessible and feasible approach to DM screening [10–12]. These systems require minimal sample volumes (1–20 µL), provide rapid results, and simplify specimen collection logistics. Some PoCT devices, such as DCA and Cobas b 101, function as benchtop analyzers based on immunoassay principles. Portable cartridge-based systems, such as A1CNow, rely on simplified analytical workflows and require minimal operator training, which enhances their applicability in decentralized or community-based settings [10, 11, 13]. Additionally, self-collection strategies using Dried Blood Spot (DBS) techniques, which involve collecting whole capillary blood on filter paper, further expanding access among hard-to-reach populations [14].

Recent systematic evidence has supported the implementation of these technologies. Gourlay et al. [15] demonstrated that PoCT devices for HbA1c improved the detection of undiagnosed DM and prediabetes, enhanced glycemic monitoring, and increased patient satisfaction. However, the aim of this review was to analyze PoCT in primary health care. Similarly, Moore et al. [16] found that PoCT strategies in population screening programs boosted coverage among underserved groups and contributed to reducing health inequities.

Given the relevance of this issue, this scoping review aimed to map the available evidence on the use of capillary blood collection technologies in the context of HbA1c screening and examine their applicability, accuracy, operational feasibility, and implementation barriers. By synthesizing existing knowledge, this review intends to offer strategic discussions to expand the reach of epidemiological surveys, strengthen public health policies, and promote more equitable approaches to DM control on a global scale.

## Materials and methods

A scoping review was undertaken to map and describe empirical studies evaluating capillary blood–based technologies for HbA1c measurement in epidemiological contexts and to identify gaps in the existing evidence. The review was conducted in accordance with JBI methodology [17] and reported following PRISMA-ScR guidelines [18]. The methods were guided by a pre-specified protocol Breternitz et al. [19] registered on the Open Science Framework (https://doi.org/10.17605/OSF.IO/39Q8T). These frameworks informed the definition of the review scope, development of eligibility criteria, search strategy, data extraction, and presentation of findings. This review aimed to answer the following research question:

*Which capillary blood–based technologies and approaches have been used for HbA1c measurement in epidemiological and field-based studies*,* considering their purposes*,* populations*,* settings*,* geographic distribution*,* country income level*,* and temporal trends?*

### Inclusion criteria

The review question and eligibility criteria were developed using the Population–Concept–Context (PCC) framework recommended by the JBI methodology for scoping reviews (Table 1). This framework guided the formulation of the review question, the development of inclusion criteria, and the organization of data extraction and synthesis.

**Table 1:** Population–concept–context (PCC) framework applied to eligibility criteria

Acronym	Inclusion Criteria
(P) Participants	Adults (≥ 18 years)
(C) Concept	Capillary blood–based technologies and approaches for HbA1c measurement
(C) Context	Epidemiological and field-based studies conducted across diverse geographic, socioeconomic, and healthcare contexts in which capillary blood HbA1c measurement was applied

### Search strategy

A comprehensive search strategy was developed to identify primary epidemiological studies assessing capillary blood–based technologies for HbA1c measurement. An initial limited search was conducted in MEDLINE (PubMed) and EMBASE to identify relevant articles on these technologies. Text words and index terms extracted from titles and abstracts were used to develop the final search strategy.

The search terms were organized according to the PCC framework. Participants (P) were defined during the study selection process and included adults aged ≥ 18 years. The Concept (C) was operationalized using the following keywords: “point-of-care testing”, “POCT”, “rapid diagnostic test”, “capillary blood”, “fingerstick”, “fingerpick”, “point of care”, “portable device”, “dried blood spot”, and “dried blood spot testing”. These terms were combined with “glycated hemoglobin”, “diabetes mellitus”, and “hyperglycemia” to ensure the identification of studies evaluating capillary-based HbA1c measurement approaches within relevant clinical contexts. The Context (C) was represented by the following search terms: “surveys and questionnaires”, “survey”, “epidemiologic methods”, and “community surveys”.

The strategy was adapted for PubMed, EMBASE, Scopus, Web of Science, LILACS, and CINAHL. No restrictions were applied regarding language, year of publication, or geographic location. The search was designed to retrieve studies indexed up to 31 January 2025 and was conducted between 18 and 24 February 2025 (Supplementary Material I).

### Study of evidence selection

The search results from each database were imported into Rayyan QCRI, where duplicate records and clearly irrelevant titles were removed. Following this initial deduplication, the first screening phase was conducted, in which the titles and abstracts of the remaining records were independently examined by two reviewers to identify potentially relevant studies, according to the predefined inclusion and exclusion criteria outlined in the protocol registered on the OSF platform. When necessary, the study conclusions were consulted to determine relevance. Discrepancies were resolved through discussion or, when required, consultation with a third reviewer.

In the second screening phase, potentially eligible records were re-evaluated with a focus on the study objectives and methodologies to ensure eligibility. Full-text articles meeting the inclusion criteria were retrieved and independently assessed by both reviewers using a standardized data extraction form.

Citation details were initially collected using a Google Forms–based extraction tool and subsequently organized in an Excel spreadsheet. Full-text sources that did not meet the eligibility criteria were excluded, and the reasons for exclusion were systematically documented (Supplementary Material III). The extraction form was piloted on a subset of studies to ensure consistency between reviewers, and all extracted data were verified for completeness prior to synthesis.

### Data extraction

A structured data extraction form was employed as a methodological tool to ensure standardization throughout the review process, allowing for systematic collection of information from the included studies. The form comprises categorical variables (such as study design, population characteristics, and type of technology), continuous variables (including sample size and age range), and qualitative data obtained through open descriptive fields. This format enabled an integrated and detailed analysis across studies, enhancing the uniformity of data cleaning, and facilitating the tabulation of findings.

The extracted information included study characteristics, population profile, type of capillary blood-based technology, application context, and outcome domains including applicability, accuracy, and feasibility. A descriptive analytical approach was employed to categorize the studies according to these attributes (see Supplementary Material II). The results are summarized in figures and complemented by a narrative synthesis that contextualizes the findings within the existing literature and discusses key methodological challenges.

### Data analysis and presentation

The findings of this scoping review are presented using a structured approach guided by the analytical questions of WHAT, WHY, WHERE, WHEN and WHO enables a comprehensive understanding of the landscape of capillary blood-based technologies for diabetes screening and monitoring. The results section is organized into five key parts: (I - WHAT) mapping analytical technologies that identify capillary blood-based approaches for HbA1c (II - WHY) classification of studies by outcome, addressing the purposes and objectives of the included studies; (III - WHERE) distribution of included studies by country income level, examining the geographic and economic contexts in which these technologies have been applied; (IV - WHEN) temporal distribution of publications, describing the evolution of the scientific output over time; and (V - WHO) characteristics of included studies, population profiles, and settings of application. This framework provides a logical organization of evidence, highlighting trends, gaps, and opportunities for future research on the applicability of capillary blood testing technologies in public health and epidemiological contexts.

## Results

### Study of evidence inclusion

**Fig. 1:**
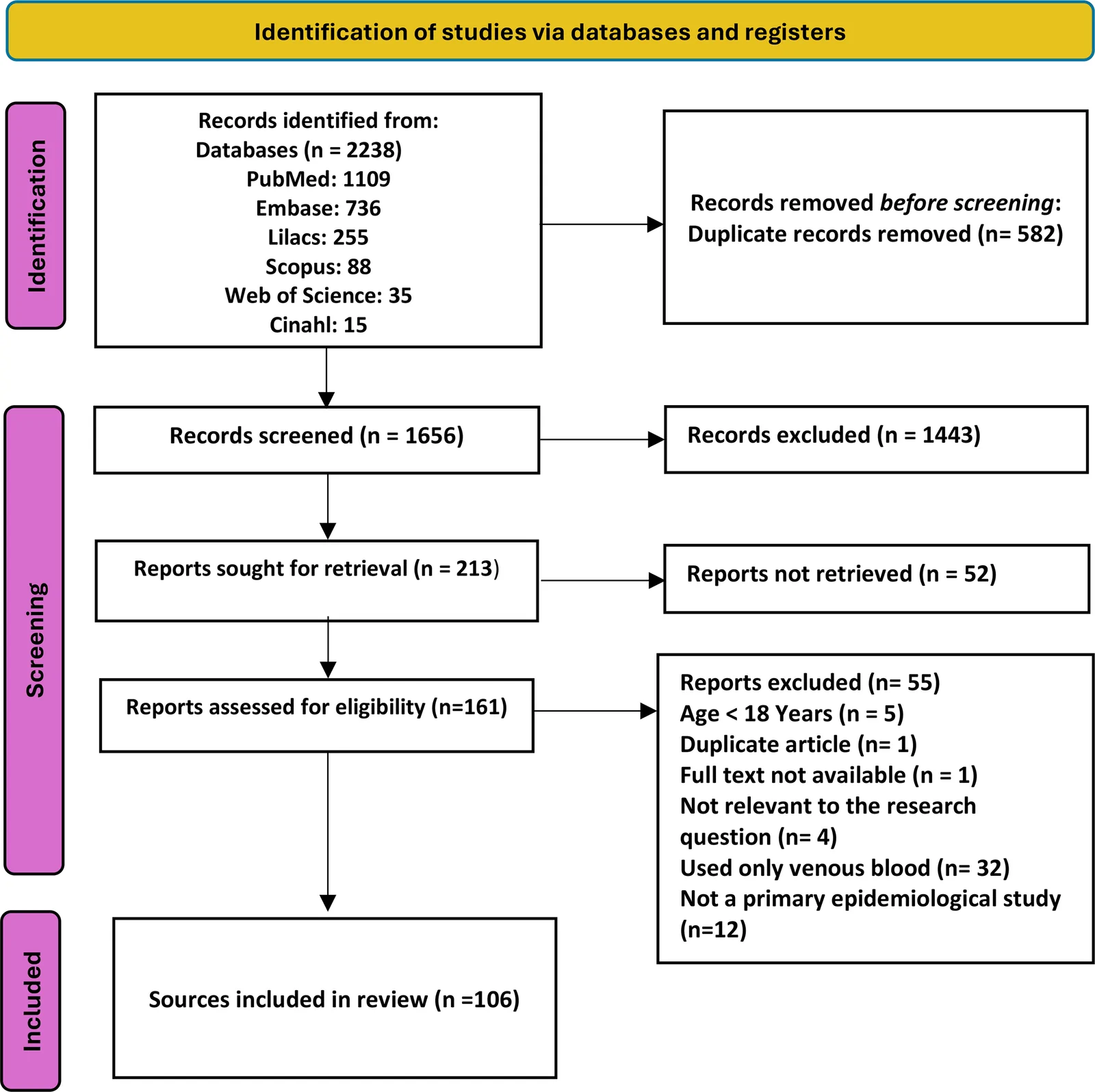
Search results and source selection and inclusion process [18]

The search identified 2,238 records, of which 582 were duplicated, leaving 1,656 records for screening in the first phase. At the title and abstract screening stage, 1,143 records were excluded as they were not aligned with the research question of this scoping review. Excluded studies included in vitro and in vivo experiments, blood bank studies without clear description of the sampling methodology, pediatric populations, and studies conducted in specific clinical contexts. A total of 213 records progressed to the second screening stage, where titles and abstracts were re-evaluated with an emphasis on study objectives and methods. The records included 161 articles qualified for full-text assessment and were independently examined by two reviewers using a standard extraction form. After this review, 55 articles were excluded because they failed to meet the eligibility criteria, leaving 106 studies for inclusion in the final analysis (Fig. 1). All extracted data were cross-checked for consistency by two reviewers.

### Mapping of analytical technologies

**Fig. 2:**
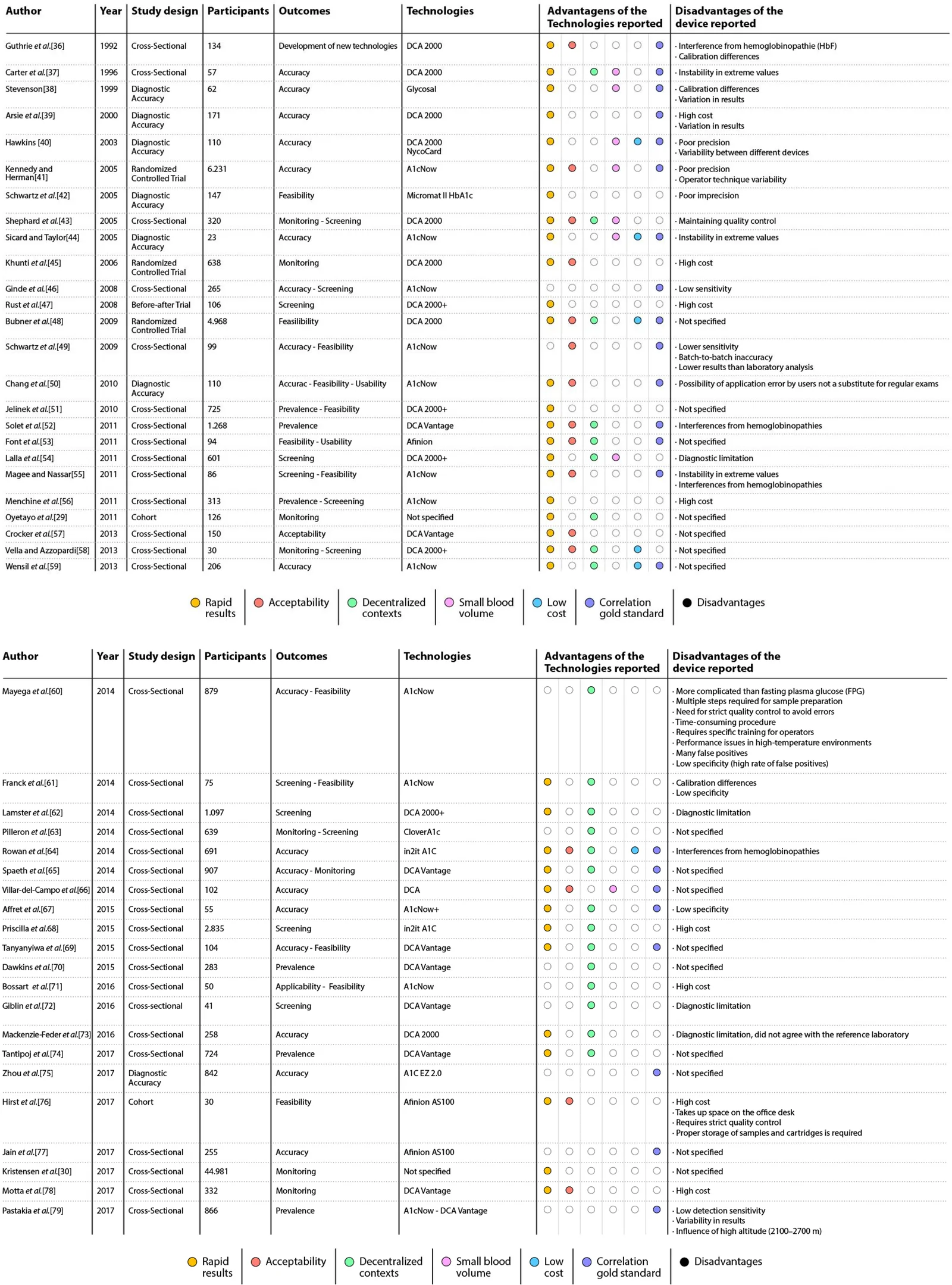

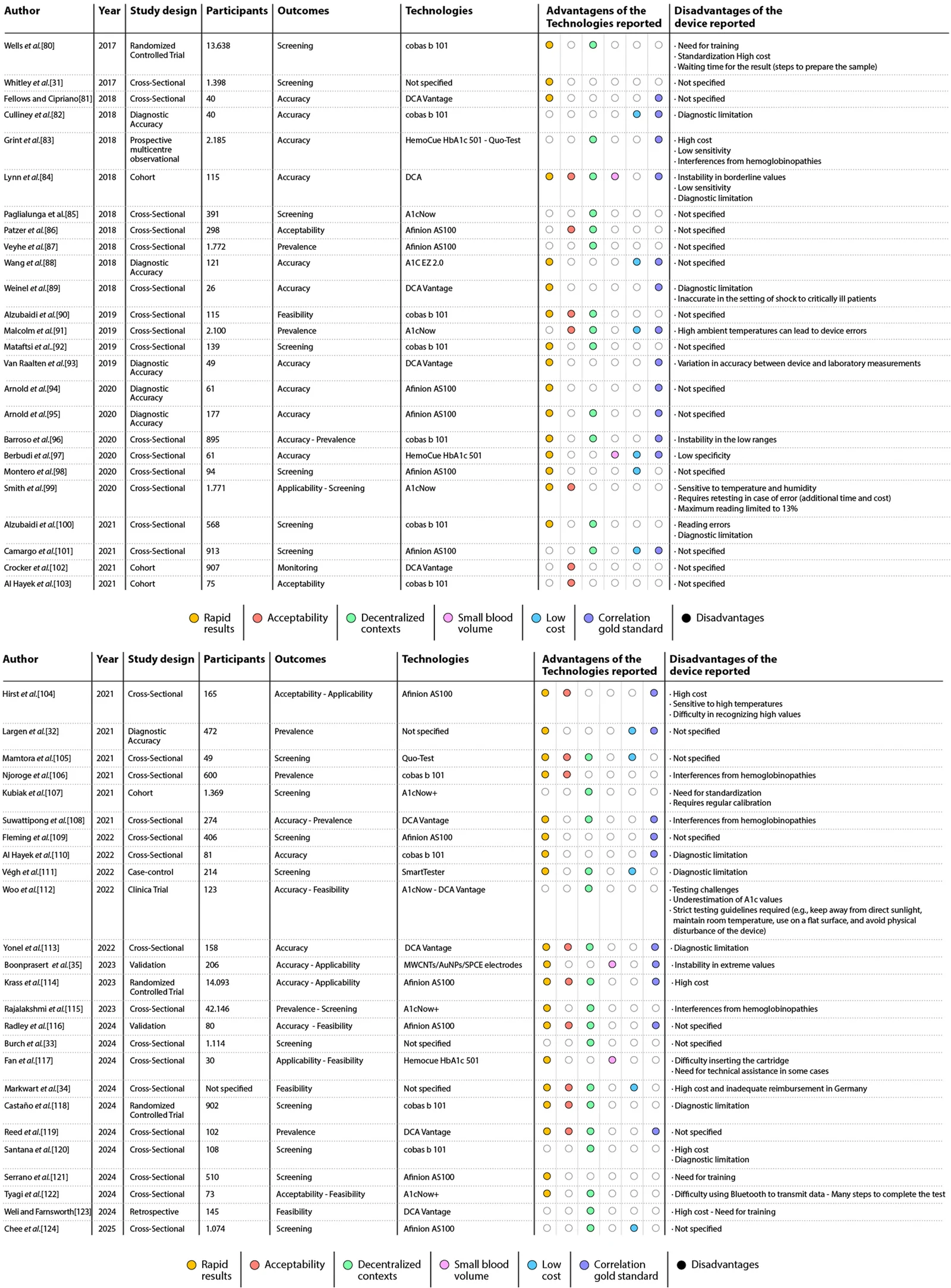
Mapping of PoCT technologies for HbA1c measurement in epidemiological studies (1992–Jan 2025)

**Fig. 3:**
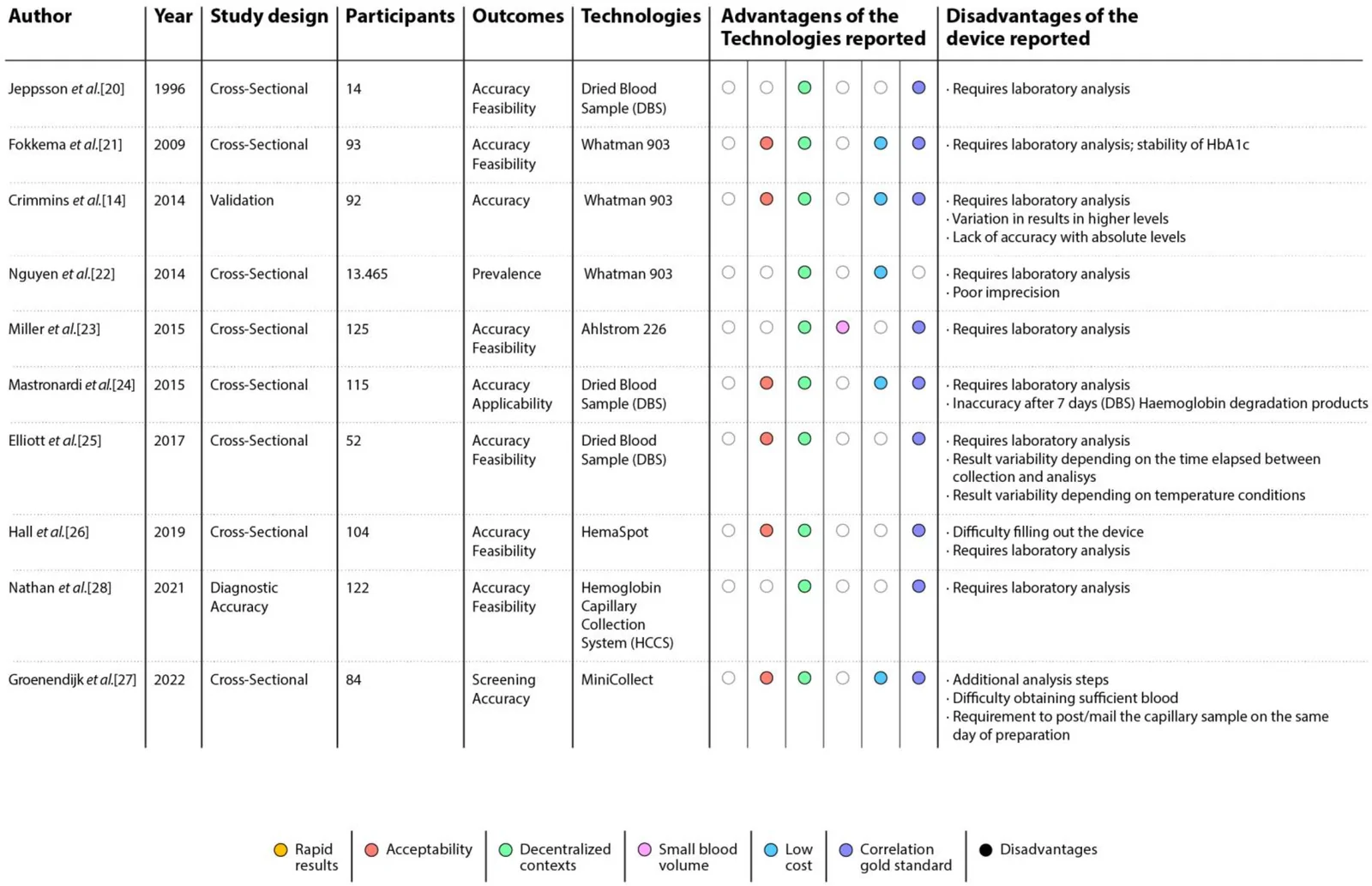
Mapping of DBS, HCCS and MiniCollect technologies for HbA1c measurement in epidemiological studies (1992–Jan 2025)

A total of 106 studies were included in this scoping review for data extraction and analysis. A total of 13 distinct PoCT technologies for HbA1c measurement and five distinct capillary blood collection and filter paper storage systems were identified. Figures 2 and 3 summarize the capillary blood-based technologies, highlighting their reported advantages and limitations, particularly regarding analytical performance and operational feasibility. Figure 2 presents portable and benchtop PoCT devices capable of providing immediate HbA1c results at the point of care [29–124], whereas Fig. 3 illustrates technologies that require subsequent laboratory analysis. The latter includes studies using DBS samples, MiniCollect^®^, and the Hemoglobin Capillary Collection System (HCCS) [14, 20–28].

Among the PoCT devices, the most frequently reported were DCA (Siemens, *n* = 33), A1CNow (PTS, *n* = 20), Afinion (Abbott, *n* = 15), and Cobas b 101 (Roche, *n* = 11), which together accounted for > 75% of all technologies identified. Some of these models represent different generations of the same device, such as Siemens’ DCA, DCA 2000, DCA 2000+, and DCA Vantage. Most PoCT devices require 1–20 µL of capillary blood and provide results in approximately few minutes (3–10 min). The small sample volume and rapid turnaround time were described in several studies as operational advantages compared with venous collection.

In contrast, DBS technologies require larger sample volumes per spot (50–75 µL) but offer logistical flexibility, enabling remote or home collection and even postal delivery. Eight studies (7.5%) reported DBS use for HbA1c assessments [14, 20–26]. Another example of technology requiring subsequent analysis is the MiniCollect^®^ [27] and HCCS a 5 µL plastic capillary tube which was applied by Nathan et al. [28].

Six studies (5.6%) did not report the device used for HbA1c measurements [29–34]. Additionally, one study has reported the development of novel biosensors. Boonprasert et al. [35] designed a portable electrochemical sensor based on multi-walled carbon nanotubes (MWCNTs) incorporated with gold nanoparticles (AuNPs), showing potential for low-cost and scalable diagnostic applications. The characteristics of the included studies are summarized in Figs. 2 and 3 [14, 20–124].

### Classification of studies by outcome

The included studies were categorized according to the outcomes explicitly defined and reported by the original authors. Several studies addressed more than one outcome domain (e.g., diagnostic accuracy and applicability), resulting in 141 outcome classifications across the 106 included studies. Each outcome was counted individually to provide a comprehensive representation of the thematic distribution of the literature. As illustrated in Figs. 2 and 3, diagnostic accuracy was the most frequently investigated outcome, reported in 46 studies (43.4% of all classifications). This was followed by screening performance (31 studies; 29.2%), feasibility (25 studies; 23.5%), and diabetes prevalence estimation (15 studies; 14.1%). Less frequently, studies focused on monitoring (nine studies; 8.4%), applicability in healthcare settings (seven studies; 6.6%), and acceptability (five studies; 4.7%), defined as the perceived satisfaction and ease of use reported by physicians, healthcare professionals, and patients. Usability was examined in two studies, and the development of new technologies was described in one study. Collectively, these findings indicate that most research efforts have focused on evaluating the diagnostic performance and implementation potential of capillary blood–based HbA1c technologies.

**Fig. 4:**
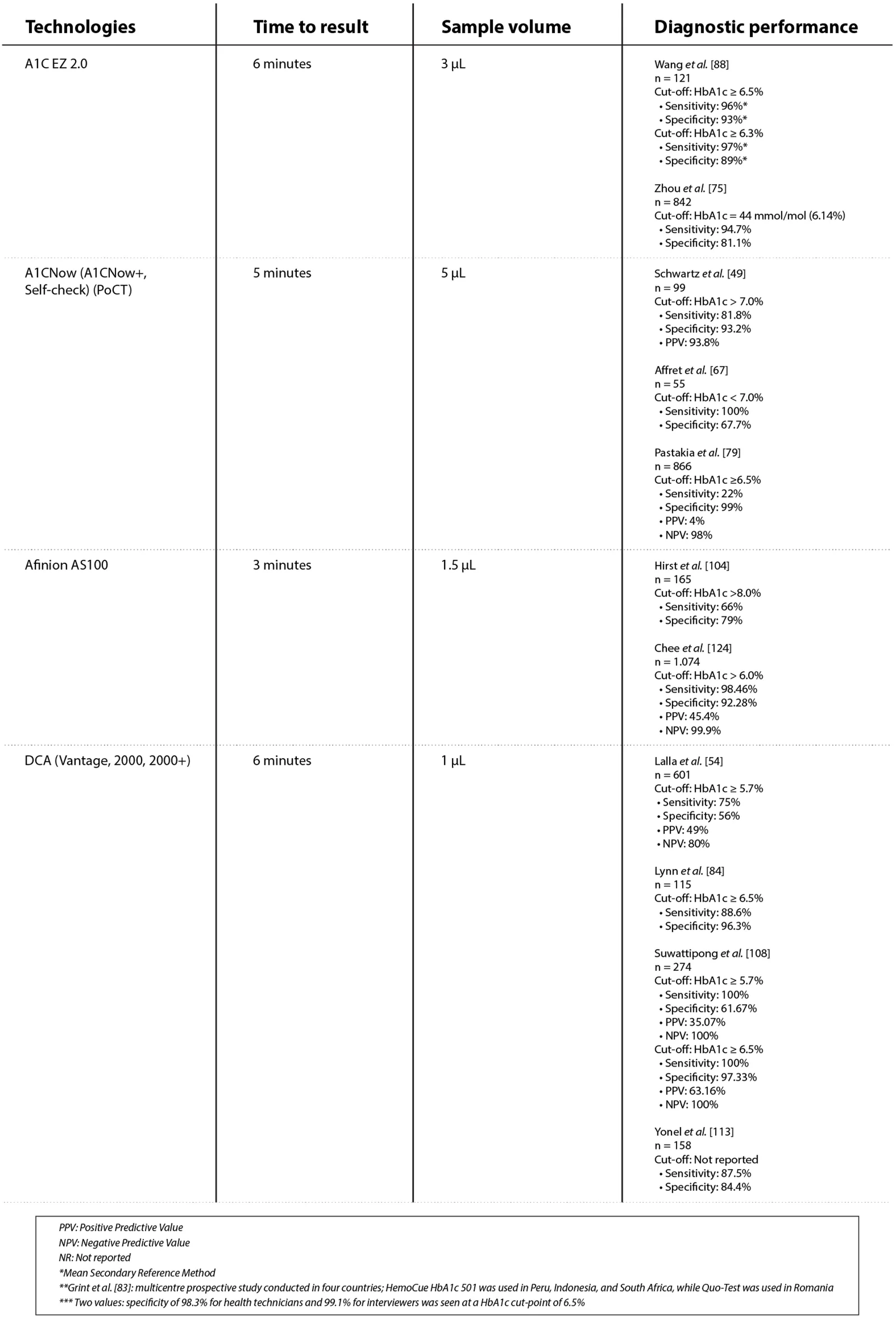

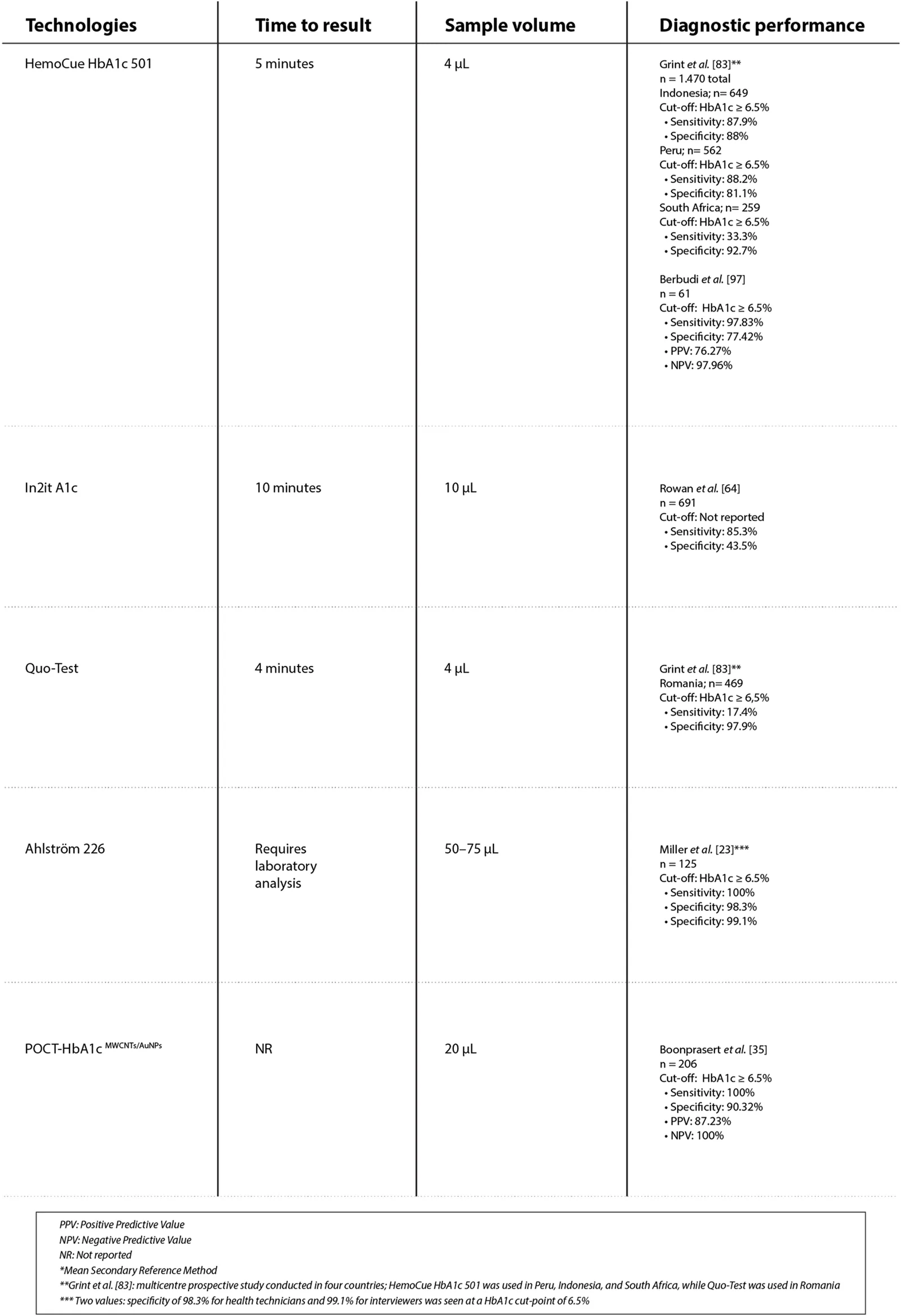
Operational characteristics and diagnostic performance of capillary blood–based HbA1c measurement technologies that reported sensitivity and specificity measures in the included studies

Despite the predominance of diagnostic accuracy studies, only 16 of 46 studies (34.8%) reporting accuracy outcomes provided complete diagnostic performance measures, including sensitivity and specificity, for capillary blood–based HbA1c technologies. This corresponds to approximately 15% of all included studies. Among the studies reporting these outcomes, sensitivity ranged from 17.4% to 100.0% while specificity ranged from 43.5% to 99.1% [23, 35, 49, 54, 64, 67, 75, 79, 83, 84, 88, 97, 104, 108, 113, 124]. These studies evaluated eight commercial technologies and one experimental prototype, with 87.5% of the identified devices being National Glycohemoglobin Standardization Program (NGSP)-certified (Fig. 4).

Among capillary blood–based HbA1c technologies, diagnostic accuracy estimates demonstrated substantial heterogeneity across studies. This variability reflects differences in study populations, clinical settings, HbA1c cut-off thresholds, reference standards, and analytical approaches. DBS-based evidence was limited to a single study Miller et al. [23]., which reported sensitivity of 100% and specificity of 98.3% for health technicians and 99.1% for interviewers, indicating consistently high diagnostic performance within this study. However, no broader comparison across DBS technologies was possible due to limited data availability.

### Distribution of included studies by country income level

**Fig. 5:**
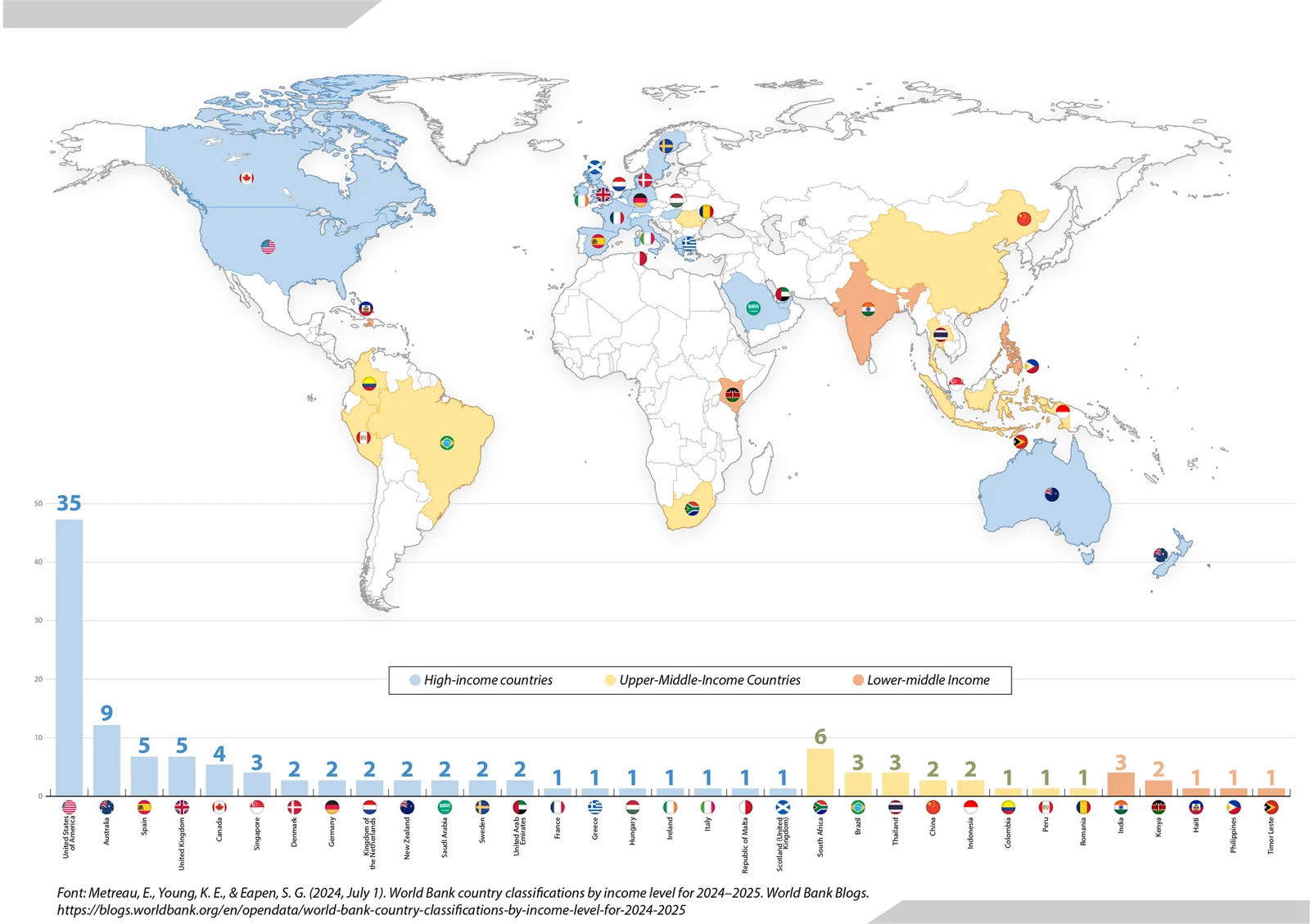
Geographic distribution of included studies by country income level (1992–Jan 2025)

Figure 5 illustrates the global distribution of studies included in this scoping review, classified by country and income level according to the World Bank’s income classification system applicable at the time of each study’s publication using the official classifications available at: https://blogs.worldbank.org/en/opendata/world-bank-country-classifications-by-income-level-for-2024-2025. For studies conducted across multiple countries, each country was counted separately in geographic distribution analysis. Only one included study [83] was multicounty, recruiting participants from Indonesia, Peru, Romania, and South Africa. As a result, the total number of study locations (*n* = 109) exceeds the total number of studies included. A total of studies were conducted across 33 countries, with the United States (*n* = 35) being the most represented among High-Income Countries (HICs) far surpassing other high-income countries, such as Australia (*n* = 9), Spain (*n* = 5), and the United Kingdom (*n* = 5). In contrast, few studies have been conducted in upper-middle- and lower-middle-income countries, including South Africa (*n* = 6), Brazil (*n* = 3), and India (*n* = 3), respectively. This pattern indicates that most of the available evidence was generated in regions with greater research infrastructure and resources, whereas contributions from resource-constrained contexts remain limited.

Studies were predominantly conducted in HICs, accounting for a substantial portion of the evidence (77%). By contrast, LMIC including upper-middle countries such as Brazil, South Africa, and India, have contributed to a smaller share of studies, highlighting a geographic imbalance in the research landscape. This asymmetry suggests that the applicability and scalability of technologies using capillary blood collection in LMICs remain underexplored in the current literature.

### Temporal distribution of publications

**Fig. 6:**
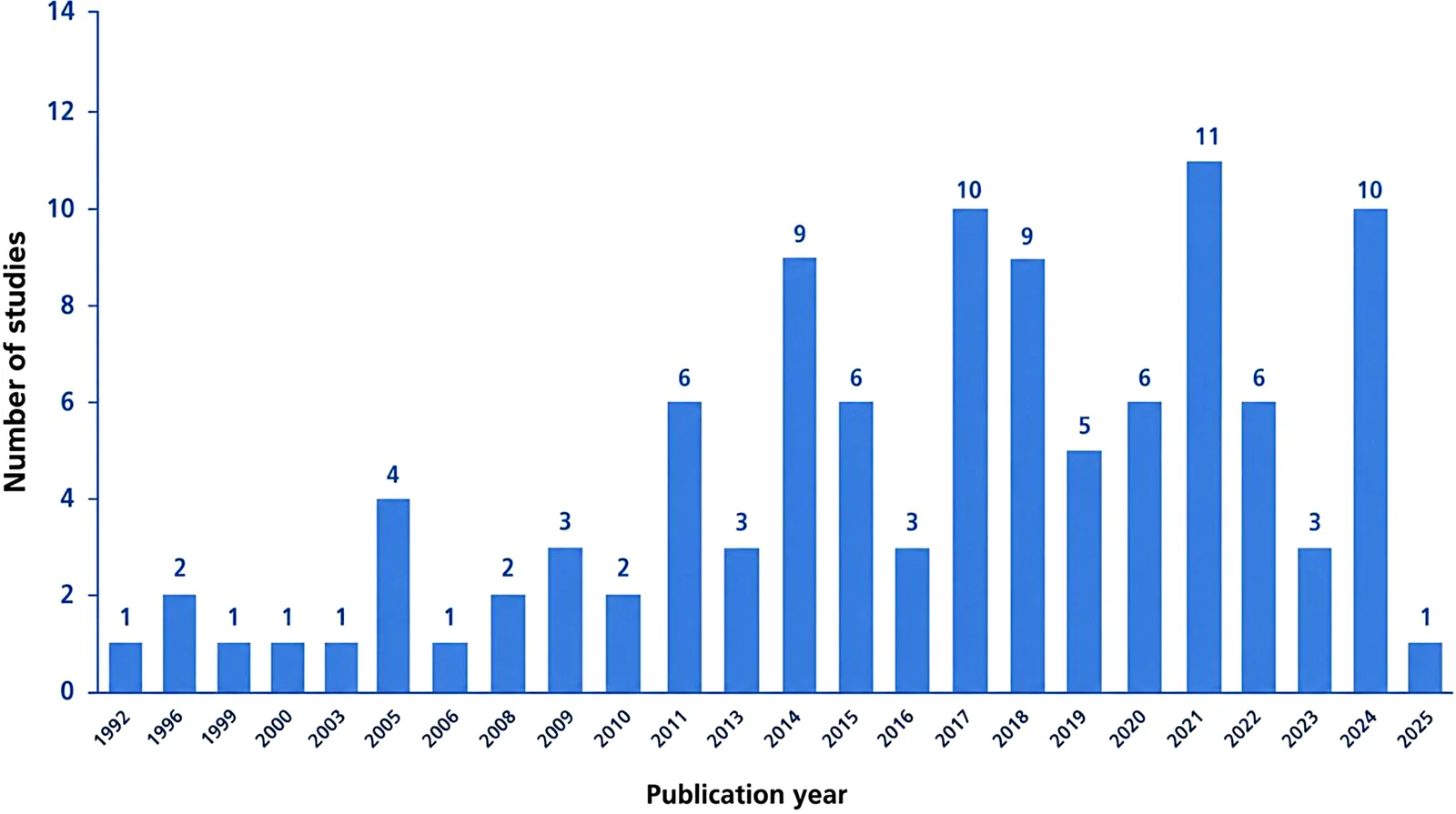
Number of included studies by year of publication (1992–Jan 2025). Note As the search process for this scoping review was completed in January 2025

Regarding the annual distribution of studies included in this scoping review, Figs. 2 and 3 display the year of publication alongside the respective authors, revealing a consistent upward trend from the first study identified in 1992 through 2025. This increase reflects the growing interest in capillary blood–based technologies for diabetes screening, likely driven by technological advances and their gradual integration into diverse healthcare and research contexts. In the past decade, this upward trend has become more evident, with 65% of the studies (*n* = 70) being published during this period. The peak years are 2017, 2021, and 2024, each with ten or more publications addressing the use of these technologies in epidemiological studies (Fig. 6). As the search for this review was completed in January 2025, only one study published that year was included.

### Characteristics of included studies

Most epidemiological and field studies employed a cross-sectional design (*n* = 73; 68.9%), followed by diagnostic accuracy studies (12.6%), cohort studies, and randomized controlled trials, each representing 5.6% (Figs. 2 and 3). There was considerable heterogeneity across the included studies with respect to the sample size. For this analysis, 105 of the 106 studies that quantitatively reported the number of participants were considered, and one study [34] reported aggregated data from 1,575 medical practices in a retrospective, consecutive cross-sectional analysis of outpatient healthcare claims (2017–2022), which could not be converted into individual participant counts. Among the 105 studies analyzed, the sample sizes ranged from 23 participants [44] to 44,981 [30]. This variability underscores the adaptability of capillary blood-based technologies across diverse study designs and contexts.

Age distribution included studies that consistently involved adult populations (*≥* 18 years). The reported age descriptors varied, with some studies providing mean or median values. Overall, the age range across the studies ranged from 18 to 97 years. The research settings were heterogeneous, with several studies reporting more than one location. The most frequently cited settings were primary healthcare units (*n* = 31), outpatient clinics (*n* = 28), and hospitals (*n* = 23). Additional contexts included research institutes (*n* = 13), home-based settings (*n* = 15), population-based surveys (*n* = 13), dental clinics (*n* = 9), and pharmacies (*n* = 8). Within hospitals, studies have been conducted in urgent care, emergency departments, and intensive care units [46, 51, 55, 56]. Vulnerable subgroups were variably represented including individuals living with HIV [106, 107], pregnant women [82, 123], those with tuberculosis [32, 83], and ophthalmological populations [70, 105] which remain underrepresented relative to broader adult cohorts.

## Discussion

Capillary blood collection technologies may represent a strategic advancement in the early detection and monitoring of levels of HbA1c, a biomarker for DM, a chronic noncommunicable disease that continues to impose a burden on global health systems. According to the GBD 2021 Diabetes Collaborators [1], DM is a major contributor to both years of lost (YLLs) and years lived with disability (YLDs), significantly reducing quality of life and straining public healthcare resources. The vast majority of the global diabetes burden, approximately 96% of total cases and 95.4% of disability-adjusted life years (DALYs) are attributed to type 2 diabetes.

In this scenario, we conducted a scoping review and synthesized the findings using a 5 W framework to provide a comprehensive view of the technologies employed, the rationale for their use, where and when they have been implemented, and the characteristics of the populations studied. [WHAT] Mapped capillary blood–based analytical technologies for HbA1c, were predominantly used in PoCT and DBS; despite the large number of studies, use was concentrated in four PoCT devices. [WHY] most investigations have assessed the diagnostic accuracy and feasibility across care settings, especially in primary care. [WHERE] applications are largely situated in HIC. [WHEN] scientific output has increased over the past decade, reflecting the growing momentum toward the decentralized use of these technologies. [WHO] study populations were mainly adults with or without diabetes or prediabetes. Beyond primary care, studies were also reported in hospitals, outpatient clinics, pharmacies, and dental practices, which emerged as screening venues for early detection. Across these contexts, reported advantages include portability, rapid turnaround, minimal sample volume, and acceptable correlation with reference methods. However, barriers include insufficient support for definitive diagnosis without laboratory confirmation, reduced precision at extreme HbA1c values, and susceptibility to hemoglobinopathies and other analytical interferences [39, 40, 44, 52, 53].

This scoping review was grounded on the premise that early diagnosis represents a pathway to prevent future complications associated with DM. Timely identification of hyperglycemia can help avert severe outcomes such as chronic kidney disease, peripheral arterial disease, and cardiovascular disorders. A systematic review by Gourlay et al. [15] reinforces this perspective, demonstrating that a 1% reduction in HbA1c levels is associated with a 37% decrease in microvascular complications. Therefore, expanding access to screening and long-term monitoring has been discussed in literature as potentially relevant for therapeutic management and patient outcomes.

Despite being the gold standard, traditional laboratory-based testing methods may be inaccessible or logistically impractical in underserved areas. In contrast, capillary blood sampling for HbA1c measurement, particularly PoCT and DBS technologies, has been a scalable alternative in settings that require rapid results and/or lack laboratory infrastructure [48, 97, 101]. These tools have been reported in the literature for use in diabetes detection, including in minoritized populations with limited access to healthcare services.

A study conducted by Moore et al. [16] highlighted that most research on capillary blood collection using PoCT focuses on evaluating clinical performance, particularly the diagnostic accuracy of these devices. Although Moore’s review was not limited to the HbA1c biomarker, but instead to PoCT applied in emergency medical services, its findings reflect a growing scientific and clinical interest in the broader potential of these technologies. This study may provide evidence on how these technologies can support clinical decision-making, reduce diagnostic delays, and improve patient management.

By mapping the available evidence on capillary blood technologies for HbA1c testing, this study identifies consistent adoption patterns that may signal market consolidation. Four devices, DCA (Siemens), A1CNow (PTS Diagnostics), Afinion (Abbott), and cobas b 101 (Roche Diagnostics), dominate epidemiological applications, accounting for 75% of the studies included. This concentration likely reflects several factors: established analytical performance, regulatory validation pathways, distribution on a large scale, and user experience, all of which favor predictability and procurement in screening programs. Approaches such as DBS and HCCS have expanded the feasibility of population-level screening in remote or decentralized contexts [14, 20–28]. DBS necessarily entails subsequent laboratory analysis and delayed result delivery, but it enables household-level sampling and postal logistics at the scale [22, 23], HCCS well as supports home sampling with HbA1c measurement, although routine clinical use still requires regulatory clearance [28].

An important finding of this scoping review was that, although diagnostic accuracy was the most frequently investigated outcome, only 16 studies reported sensitivity, and specificity estimates for capillary blood–based HbA1c technologies. Moreover, substantial variability was observed in the reported diagnostic performance, likely reflecting differences in study populations, HbA1c cut-off values, reference methods, clinical settings, and device characteristics. Notably, many studies assessed analytical performance using agreement-based approaches, such as correlation analyses, concordance measures, and method-comparison techniques, rather than diagnostic accuracy metrics. These findings are consistent with the systematic review and meta-analysis by Hirst et al. [125], which identified considerable methodological heterogeneity among studies evaluating HbA1c PoCT devices and highlighted the inconsistent reporting of standardized diagnostic performance measures. Collectively, these observations indicate that direct comparisons of diagnostic accuracy across technologies should be interpreted with caution and underscore the need for standardized evaluation frameworks to support the validation and implementation of capillary blood–based HbA1c testing.

Although 77% of the 106 included studies were conducted in HIC settings, the burden of chronic diseases is rising fastest in LMIC. According to the International Diabetes Federation Diabetes Atlas: 11th edition [126], it is estimated that a 95% increase in the number of individuals with diabetes by 2050 will occur in low- and middle-income countries. In this sense, early detection of diabetes and timely initiation of treatment may be decisive for the effective management and prevention of complications. The longer a person lives with undiagnosed diabetes, the greater the risk of developing complications [8]. Strengthening primary health care with integrated screening strategies is important to detect noncommunicable diseases [127], and our review shows that primary care is the most common implementation venue (*n* = 31). In these settings, PoCT for HbA1c can be cost-effective relative to laboratory testing, improving access and potentially lowering downstream costs with future complications when embedded in workflows with appropriate quality control [128].

The advantages reported across studies included low sample volume requirements, rapid turnaround times for PoCT devices, high patient acceptability, and, in many cases, acceptable agreement with reference laboratory methods. However, limitations related to interference from hemoglobin variants have been consistently reported over time. These limitations were already described in the earliest study included in this review by Guthrie et al. [36] and have persisted across successive technological generations of capillary blood-based HbA1c measurement systems [55, 64, 83, 106, 108, 115], indicating that they are not restricted to early-generation technologies but represent ongoing analytical challenges.

Immunoassay-based techniques, commonly used in several PoCT devices, rely on antigen–antibody interactions and may be more susceptible to these interferences. This is particularly relevant for handheld disposable systems such as A1CNow, which employ immunoassay principles. In contrast, the Afinion system, a benchtop PoCT analyzer based on boronate affinity chromatography, has not been associated with interference from hemoglobin variants in the included studies. For DBS-based approaches, including HCCS and MiniCollect systems, sample analysis is not performed at the point of collection but is subsequently conducted in centralized laboratory settings. It is important to emphasize that these sources of analytical error are not restricted to early-generation technologies but reflect both historical and persistent limitations across the evolution of HbA1c measurement systems and may introduce bias in results [39, 40, 44, 52, 53].

In addition, accuracy-related limitations may vary at extreme glycemic ranges [35, 37, 44, 55], and agreement with laboratory standards may differ across devices and settings, with reports of batch-to-batch variability and calibration drift. These findings reinforce that PoCT should not replace confirmatory laboratory testing for definitive diagnosis [34, 41, 49]. Environmental factors such as heat, reagent handling, and deviations from manufacturer-recommended quality control protocols further compromise performance, mainly for DBS shipping and external laboratory processing [21, 24, 91, 99, 104].

In rural Brazil, a two-step strategy (health fairs and patient groups) has demonstrated the feasibility of routine use of these technologies and meaningful community engagement [101]. Pharmacies and dental clinics have emerged as additional resources for screening of the HbA1c and health education, with triage effectiveness and cost–benefit as complementary public-health strategies [54, 74, 120].

The epidemiological populations studied were heterogeneous spanning people with comorbidities such as individuals living with HIV [106, 107], pregnant [82, 123], and those with tuberculosis [32, 83]. This demonstrates the potential of these technologies, while emphasizing the need to adapt device selection, infrastructure, and follow-up pathways to local clinical contexts.

Among the limitations reported by the studies regarding the use of these devices are differences in standardization across reference methods and the lack of direct alignment with international standards, such as the NGSP, which may undermine external comparability [36, 38, 41, 42]. Epidemiological studies relied on small samples, and the lack of test replication and operator heterogeneity led to variance and random error [37, 41, 49, 84]. Consumer costs (cartridges/reagents), ongoing training needs, and the requirement for standardized workflows challenge scalability and financial sustainability beyond research environments [43, 47, 61, 78, 99, 116, 120].

Despite system-integration efforts to mitigate equity gaps, data generated at triage points in pharmacies and dental clinics do not guarantee return rates for confirmatory testing or continuity of care; narrow demographic samples and regional constraints further restrict extrapolation [22, 30, 34, 54, 86, 87, 90]. Accordingly, gains will depend on coupling analytical performance, accuracy, structural and economic viability, and acceptability with context-specific implementation and integration of these technologies for HbA1c yield measurable improvements in screening coverage, timely diagnosis, and appropriate follow-up, particularly in LMIC settings.

Consistent with JBI guidance for scoping reviews [17], we did not perform a formal critical appraisal of individual studies or apply GRADE to assess the certainty of evidence, as our objective was to map the breadth of available research. We also excluded bench-only validation studies without population data, focusing instead on clinical and epidemiological applications. Secondary syntheses and gray literature (e.g., technical reports, dissertations, conference abstracts) were not included to concentrate on peer-reviewed studies, thereby ensuring methodological standardization, metadata consistency, and reproducibility of the search. Considerable heterogeneity across study settings, reference standards, and outcome definitions precluded quantitative synthesis and limited direct cross-study comparisons.

## Conclusions

A scoping review mapped available evidence on capillary blood–based technologies for Hba1c screening in epidemiological studies. A total of 106 studies were included and 15 distinct technologies for HbA1c measurement were identified. The most frequently reported PoCT devices, wich demonstrate advantages such as requiring a small blood volume and providing rapid results. Although represented in few studies, DBS technology seems to have important logistical benefits, enabling remote and decentralized collection with postal shipment to reference laboratories. The most frequently assessed outcomes across capillary blood–based technologies were accuracy, screening performance, and feasibility, highlighting the ongoing efforts in the analytical validation and application of these tools. However, few studies have reported complete estimates of sensitivity and specificity, and only a minority examined the feasibility, acceptability, or applicability of population-based surveys. The literature showed a clear geographic imbalance, with validation studies and outcome assessments concentrated in HIC, while evidence from LMIC countries remains scarce. This review fosters new research aimed at applying these findings to population-based surveys and broader studies on capillary blood technologies.

## Supplementary Information

Below is the link to the electronic supplementary material.

Supplementary Material 1

## Data Availability

All data analysed during this study are derived from published studies and are included in this published article and its supplementary information files. No primary datasets were generated.
