# Supplementary material for: HbA1c measurement using capillary blood in epidemiological studies: a scoping review of technologies and approaches
Source: BMC Endocr Disord. 2026 Jul 11;26:307. doi: 10.1186/s12902-026-02410-y (PMC13644120; doi:10.1186/s12902-026-02410-y)
Supplement: Supplementary file 1 [file 12902_2026_2410_MOESM1_ESM.docx]

# **Supplementary Material I: Search strategy**

| Search conducted: between 18 and 24 February 2025. | | |
| --- | --- | --- |
| **Search** | **Query** | **Records retrieved** |
| **PubMed** | (("Point of care testing"[MeSH Terms] OR "Point of care testing"[Text Word] OR "Rapid Diagnostic Test"[Text Word] OR "Capillary blood"[Text Word] OR "Fingerstick"[Text Word] OR "Fingerpick"[Text Word] OR "Point of care"[Text Word] OR "POCT"[Text Word] OR "Portable device"[Text Word] OR "Dried blood spot"[Text Word] OR "Dried blood spot testing"[Text Word]) AND ("diabetes mellitus/diagnosis"[MeSH Terms] OR "Diabetes mellitus diagnosis"[Text Word] OR "Glycated Hemoglobin"[MeSH Terms] OR "Glycated Hemoglobin"[Text Word] OR "Hyperglycemia"[Text Word] OR "Hyperglycemia"[MeSH Terms]) AND ("surveys and questionnaires"[MeSH Terms] OR "survey*"[Text Word] OR "epidemiologic methods"[MeSH Terms] OR "Community Surveys"[Text Word])) NOT ("ultrasonography"[MeSH Terms] OR "ultrasonography"[Text Word] OR "ultrasound"[Text Word]) | **1109** |
| **Embase** | ('point of care testing'/exp OR 'point of care testing':ti,ab OR 'rapid diagnostic test':ti,ab OR 'capillary blood':ti,ab OR 'fingerstick':ti,ab OR 'fingerpick':ti,ab OR 'point of care':ti,ab OR poct:ti,ab OR 'portable device':ti,ab OR 'dried blood spot':ti,ab OR 'dried blood spot testing':ti,ab) AND ('diabetes mellitus'/exp OR 'diabetes mellitus':ti,ab OR 'glycated hemoglobin'/exp OR 'glycated hemoglobin':ti,ab OR 'hyperglycemia'/exp OR 'hyperglycemia':ti,ab) AND ('questionnaire'/exp OR survey*:ti,ab OR 'epidemiologic methods'/exp OR 'community surveys':ti,ab) NOT ('ultrasonography'/exp OR ultrasonography:ti,ab OR ultrasound:ti,ab) AND [article]/lim AND ([adult]/lim OR [young adult]/lim OR [middle aged]/lim OR [aged]/lim OR [very elderly]/lim) AND [humans]/lim AND [abstracts]/lim AND [clinical study]/lim AND ([embase]/lim OR [medline]/lim OR [pubmed-not-medline]/lim) | 736 |
| **Lilacs** | ((tw:("Point-of-care testing" OR "Point of care testing" OR "Rapid Diagnostic Test" OR "Capillary blood" OR "Fingerstick" OR "Fingerpick" OR "Point of care" OR "POCT" OR "Portable device" OR "Dried blood spot" OR "Dried blood spot testing"))) AND ((tw:("Diabetes mellitus diagnosis" OR "Glycated Hemoglobin" OR "Hyperglycemia"))) AND ((tw:("Surveys and Questionnaires" OR "Survey*" OR "Epidemiologic Methods" OR "Community Surveys"))) AND NOT ((tw:("Ultrasonography" OR "Ultrasound"))) | **255** |
| **Scopus** | (TITLE-ABS-KEY ("Point-of-care testing" OR "Point of care testing" OR "Rapid Diagnostic Test" OR "Capillary blood" OR "Fingerstick" OR "Fingerpick" OR "Point of care" OR "POCT" OR "Portable device" OR "Dried blood spot" OR "Dried blood spot testing")) AND (TITLE-ABS-KEY ("Diabetes mellitus diagnosis" OR "Glycated Hemoglobin" OR "Hyperglycemia")) AND (TITLE-ABS-KEY ("Surveys and Questionnaires" OR "Survey*" OR "Epidemiologic Methods" OR "Community Surveys")) AND NOT (TITLE-ABS-KEY ("Ultrasonography" OR "Ultrasound")) | **88** |
| **Web of Science** | TS=("point-of-care testing" OR "rapid diagnostic test" OR "capillary blood" OR "fingerstick" OR "fingerpick" OR "point of care" OR POCT OR "portable device" OR "dried blood spot" OR "dried blood spot testing") AND TS=("diabetes mellitus diagnosis" OR "glycated hemoglobin" OR "hyperglycemia") AND TS=("surveys and questionnaires" OR survey* OR "epidemiologic methods" OR "community surveys") NOT TS=(ultrasonography OR ultrasonography OR ultrasound) | **35** |
| **Cinahl** | ((MH "Point-of-Care Testing" OR "Point of care testing" OR "Rapid Diagnostic Test" OR "Capillary blood" OR "Fingerstick" OR "Fingerpick" OR "Point of care" OR POCT OR "Portable device" OR "Dried blood spot" OR "Dried blood spot testing")) AND ((MH "Diabetes Mellitus, Diagnosis" OR "Diabetes mellitus diagnosis" OR MH "Glycated Hemoglobin" OR "Glycated Hemoglobin" OR MH "Hyperglycemia" OR "Hyperglycemia")) AND ((MH "Surveys and Questionnaires" OR Survey* OR MH "Epidemiologic Methods" OR "Community Surveys")) NOT ((MH "Ultrasonography" OR ultrasonography OR ultrasound)) | **15** |

# **Supplementary Material II: Data extraction instrument**

Data extraction framework and key characteristics recorded

| **Field** | **Main characteristics** |
| --- | --- |
| Authors | Identify the study and summarize its outcome-based objective |
| Year of publication |  |
| Study title |  |
| Outcome-based objective |  |
| Publication type |  |
| Study design |  |
| Population characteristics | Summarize the study population by stating the age range, identifying the target group (e.g., people with specific comorbidities, pregnant, or older adults), and indicating diabetes status |
| Age range of participants |  |
| Number of participants |  |
| Diabetes type |  |
| Capillary-blood technology type | Record the capillary-blood technology, device/model, key accuracy metrics (when reported), applied setting (clinic, pharmacy, community, or home), country, and screening method used |
| Device |  |
| Accuracy |  |
| Study setting |  |
| Country |  |
| Screening method used |  |
| Rapid test performed |  |
| Device advantages/disadvantages | Identify each device’s reported advantages and disadvantages; the key implementation facilitators, user acceptance, training requirements, and operational costs; and the principal research barriers, including sample instability, transport logistics, costs, and regulatory or ethical constraints |
| Facilitators (applicability and feasibility) |  |
| Research barriers |  |

# **Supplementary Material III: Sources ineligible following full-text review**

1. Qin S, Li Z, Zhang S, et al. Point-of-care testing HbA1c screening for type 2 diabetes in urban and rural areas of China: a cost-effectiveness analysis. Front Public Health. 2024; 12:1438945.

*Reason for exclusion*: Not relevant to the research question.

2. Zhu R, Yu M, Chen S, et al. Improvement in the quality of HbA1c determination by using commutable specimens with IFCC-assigned values. Lab Med. 2017;48(2):148-153.

*Reason for exclusion*: Not relevant to the research question.

3. Friedman SD, et al. Point-of-care HbA1c screening predicts diabetic status of dental patients.  Diabetes Care. 2014;37(12):3330-3336.

*Reason for exclusion*: Not relevant to the research question.

4. Wided A, Rihab A, Meriem R, Noureddine A, Faten C. Sustained metabolic dysregulation and the emergence of diabetes: associations between HbA1c and metabolic syndrome components in Tunisian diabetic and nondiabetic groups. J Physiol Anthropol. 2024;43(1):18.

*Reason for exclusion*: Not relevant to the research question.

5. Forhan RE, Hadden WC, Harris MI, et al. Glycosylated hemoglobin determination from capillary blood samples. Utility in an epidemiologic survey of diabetes. Am J Epidemiol. 1984;119(2):159-166.

*Reason for exclusion*: Age < 18 Years*.*

6. Zeng Y, Wu J, Wang D, et al. Performance evaluation of the glycated hemoglobin A1c analyzer for point-of-care testing compared with laboratory-based devices: a multicenter validation study. Diabet Med. 2024;41(5): e16045.

*Reason for exclusion*: Age < 18 Years*.*

7. Shahim B, Wallander M, Lind M, Nyström T. Accuracy of point-of-care equipment for glucose measurement in screening for dysglycemia in patients with coronary artery disease. Diabetes Technol Ther. 2018;20(9):596-602.

*Reason for exclusion*: Age < 18 Years*.*

8. Santos AT, Salcedo E, Vasquez S, Sanchez JF. Assessment of two diabetes point-of-care analyzers measuring hemoglobin A1c in the Peruvian Amazon. Ann Glob Health. 2018;84(4):618-624.

*Reason for exclusion*: Age < 18 Years*.*

9. Cross J, Sharma S, John WG, Rayman G. Validation and feasibility of a postal system for remote monitoring of HbA1c. BMJ Open Diabetes Res Care. 2021;9(2): e002527.

*Reason for exclusion*: Age < 18 Years*.*

10. Villar-del-Campo M, Pérez-Corral J, Torres M, et al. Concordancia diagnóstica entre dos métodos de detección de hemoglobina glucosilada A1c en atención primaria. SEMERGEN. 2014;40(8):431-435.

*Reason for exclusion*: Duplicate article.

11. Kröger HP, Padberg FD, Bender BE, Fischer VD, Siebler J, Lorenz C. Evaluation of a new method for determining glycated hemoglobin with monoclonal antibodies (DCA 2000). Clin Chim Acta. 1996;254(2):95-104.

*Reason for exclusion*: Full text not available.

12. Zhao X, Li S, Zhang W, et al. Performance of an A1C and fasting capillary blood glucose test for screening newly diagnosed diabetes and pre-diabetes defined by an oral glucose tolerance test in Qingdao, China. Diabetes Res Clin Pract. 2010;88(1):29-36.

*Reason for exclusion*: *Used only venous blood.*

13. Wang Y, Liu J, Wang Z, et al. Evaluation of hemoglobin A1c measurement from filter paper using high-performance liquid chromatography and immunoturbidimetric assay. Scand J Clin Lab Invest. 2017;77(2):104-108.

*Reason for exclusion*: *Used only venous blood.*

14. Hofmann H, Schernthaner G, Schernthaner G Jr, et al. Undiagnosed diabetes mellitus and metabolic control assessed by HbA1c among residents of nursing homes. Exp Clin Endocrinol Diabetes. 2001;109(6):326-329.

*Reason for exclusion*: *Used only venous blood.*

15. Dubach IL, Christ ER, Diem P. HbA1c-testing: Evaluation of two point-of-care analysers. Prim Care Diabetes. 2019;13(6):583-587.

*Reason for exclusion*: *Used only venous blood.*

16. Hu X, Gao J, Fang J, et al. Validation and modification of dried blood spot-based glycosylated hemoglobin assay for the longitudinal aging study in India. Clin Chim Acta. 2015; 445:1-6.

*Reason for exclusion*: *Used only venous blood.*

17. Huangfu P, Kumar AMV, Sahu S, et al. Point-of-care HbA1c level for diabetes mellitus management and its accuracy among tuberculosis patients: a study in four countries. BMC Med. 2019;17(1):82.

*Reason for exclusion*: *Used only venous blood.*

18. John WG, Gledhill K, Jones M, et al. Nurse-based evaluation of point-of-care assays for glycated haemoglobin. Diabet Med. 2006;23(9):1032-1035.

*Reason for exclusion*: *Used only venous blood.*

19. Jones TG, O'Gorman C, et al. Analysis of hemoglobin A1c from dried blood spot samples with the Tina-quant II immunoturbidimetric method. J Diabetes Sci Technol. 2010;4(2):382-388.

*Reason for exclusion*: *Used only venous blood.*

20. Lakshmy R, Prabhakaran D, Reddy KS, et al. Measurement of glycated hemoglobin A1c from dried blood by turbidimetric immunoassay. J Diabetes Sci Technol. 2009;3(5):1203-1206. *Reason for exclusion*: *Used only venous blood.*
21. Ansu-Mensah M, Ginindza TG, Amponsah SK, Amponsah-Dacosta K, Aheto JM. Geographical access to point-of-care diagnostic tests for diabetes, anaemia, hepatitis B, and human immunodeficiency virus in the Bono Region, Ghana. BMC Health Serv Res. 2024; 24:1303.

*Reason for exclusion*: *Used only venous blood.*

22. Lawson L, Muc M, Oladimeji O, Iweha C, Opoola B, Abdurhaman ST, et al. Tuberculosis and diabetes in Nigerian patients with and without HIV. Int J Infect Dis. 2017; 61:121-125.

*Reason for exclusion*: *Used only venous blood.*

23. Lee J, Han CH, Lee D, Hong J, Park H. Performance evaluation of SD A1cCare as a HbA1c analyzer for point-of-care testing. Ann Lab Med. 2015;35(2):205-209.

*Reason for exclusion*: *Used only venous blood.*

24. Lenters-Westra E, English E. Are hemoglobin A1c point-of-care analyzers fit for purpose? The story continues. Clin Chem Lab Med. 2021;59(8):1291-1295.

*Reason for exclusion*: *Used only venous blood.*

25. Lenters-Westra E, Slingerland RJ. Three of seven hemoglobin A1c point-of-care instruments do not meet generally accepted analytical performance criteria. Clin Chem. 2014;60(8):1062-1072.

*Reason for exclusion*: *Used only venous blood.*

26. Maleska V, Paskal B, Wierusz-Wysocka B, Zozulinska-Ziólkiewicz D, Marciniak M. Comparison of HbA1c detection in whole blood and dried blood spots using an automated ion-exchange HPLC system. Clin Biochem. 2017;50(9):531-534.

*Reason for exclusion*: *Used only venous blood.*

27. Mcebula S, Naidoo P, Ngubane S, Omodara O, Mthiyane T, Omar A. Diabetes and abnormal glucose tolerance in subjects with tuberculosis in a South African urban center. S Afr Med J. 2017;107(8):669-673.

*Reason for exclusion*: *Used only venous blood.*

28. Paknikar S, Sloop T, Varma M, Mistry S, Singh S. Long-term performance of point-of-care hemoglobin A1c assays. J Diabetes Sci Technol. 2016;10(6):1317-1323.

*Reason for exclusion*: *Used only venous blood.*

29. Petersen JR, Ghaffari G, Tritz J, Cunnane S, Lammey M. Hemoglobin A1c: assessment of three point-of-care analyzers relative to a central laboratory method. Am J Clin Pathol. 2010;134(1):95-100.

*Reason for exclusion*: *Used only venous blood.*

30. Sølvik UO, Røysland K, Mæland A, Sandvik H, Steiro F, Thoresen M. Diagnosing diabetes mellitus: performance of hemoglobin A1c point-of-care instruments in general practice offices. Scand J Prim Health Care. 2013;31(2):84-90.

*Reason for exclusion*: *Used only venous blood.*

31. Weli K, Farnsworth T. Implementing point-of-care hemoglobin A1c testing in an obstetrics outpatient clinic. J Obstet Gynaecol Res. 2024;50(4):567-573.

*Reason for exclusion*: *Used only venous blood.*

32. Tollånes MC, Røysland K, Bøe SA, Sølvik UO, Mæland A, Sandvik H, et al. Availability and analytical quality of hemoglobin A1c point-of-care testing in general practitioners’ offices are associated with better glycemic control in type 2 diabetes. Diabet Med. 2020;37(12):2048-2055.

*Reason for exclusion*: *Used only venous blood.*

33. Veigne C, Defrance C, Vasse M, Nivet A, Dargnies O. Study of the effect of altitude on the measurement of glycated haemoglobin using point-of-care instruments. Clin Biochem. 2015;48(12):791-793.

*Reason for exclusion*: *Used only venous blood.*

34. Stavelin A. Performance of Afinion HbA1c measurements in general practice as judged by external quality assurance data. Scand J Clin Lab Invest. 2019;79(1-2):67-71.

*Reason for exclusion*: *Used only venous blood.*

35. Andersen JW, Laustsen S, Fenger-Grøn M, Vestergaard LD, Laursen AH, Guldberg R. Use of point-of-care HbA1c measurement to estimate the level of undiagnosed diabetes mellitus among 67-year-old participants in a cardiovascular screening programme in the municipality of Viborg, Denmark. Scand J Prim Health Care. 2018;36(1):77-82.

*Reason for exclusion*: *Used only venous blood.*

36. Byashalira KC, Kavishe B, Ndibalema A, Mfinanga S, Majige E, Kilonzo S. Point-of-care glycated hemoglobin A1c testing for the identification of hyperglycemia severity among individuals with dual tuberculosis and diabetes mellitus in Tanzania. BMC Endocr Disord. 2023;23(1):35. *Reason for exclusion*: Used only venous blood.
37. Zin RMWM, Haniza B, Wan Zaidah A, Mohd Yusof R, Wan Nazaimoon WM. Haemoglobin A1c: comparing performance of two point-of-care devices with laboratory analyser. Malays J Pathol. 2013;35(1):49-54. PMID: 23899208.

*Reason for exclusion*: Used only venous blood.

38. Torregrosa ME, Fernández-Alvarez JM, Perea M, Poca M, Alcaide E. Accuracy of three hemoglobin A1c point-of-care systems for glucose monitoring in patients with diabetes mellitus. Clin Chim Acta. 2015; 448:175-181.

*Reason for exclusion*: Used only venous blood.

39. Sobolesky PM, Bruns DE, Trefry T, Reitz A, Zander J, Lupsa D, et al. Multicenter assessment of a hemoglobin A1c point-of-care device for diagnosis of diabetes mellitus. J Diabetes Sci Technol. 2018;12(5):890-896.

*Reason for exclusion*: Used only venous blood.

40. Solvik UO, Røysland K, Mæland A, Sandvik H, Bøe SA. Quality control of Norwegian pharmacy HbA1c testing: a modest beginning. Scand J Prim Health Care. 2018;36(2):199-202.

*Reason for exclusion*: Used only venous blood.

41. Ejilemele A, Unabia J, Ju H, Petersen JR. A1c Gear: Laboratory quality HbA1c measurement at the point of care. Clin Chim Acta. 2015; 445:139-42.

*Reason for exclusion*: Used only venous blood.

42. Szymezak J, Leroy N, Lavalard E, Gillery P. Evaluation of the DCA Vantage analyzer for HbA1c assay. Clin Chem Lab Med. 2008;46(8):1195-8.

*Reason for exclusion*: Used only venous blood.

43. Sánchez-Mora C, Rodríguez-Oliva M, Fernández-Riejos P, Mateo J, Polo-Padillo J, Goberna R, et al. Evaluation of two HbA1c point-of-care analyzers. Clin Chem Lab Med. 2011;49(4):653-7. *Reason for exclusion*: Used only venous blood.
44. Shephard MDS, Whiting MJ. Assessment of the practicability and analytical performance of a point-of-care affinity chromatography haemoglobin A1c analyser for use in the non-laboratory setting. Ann Clin Biochem. 2006;43(6):513-520.

*Reason for exclusion*: Not a primary epidemiological study.

45. Plüddemann A, Heneghan C, Mahtani K, Onakpoya I. Primary care diagnostic technology update: point-of-care testing for glycosylated haemoglobin. Br J Gen Pract. 2011;61(583): e736-738.

*Reason for exclusion*: Not a primary epidemiological study.

46. Lalla E, Cheng B, Kunzel C, Allareddy V, Lamster IB, Glick M. Dental findings and identification of undiagnosed hyperglycemia. J Dent Res. 2013;92(2):135-140.

*Reason for exclusion*: Not a primary epidemiological study.

47. Vijan S, Fagerlin A. Diabetes risk assessment and glycemic control. J Gen Intern Med. 2015;30(5):612-618.

*Reason for exclusion*: Not a primary epidemiological study.

48. Moon J, Heo YJ, Park I, Bae S, Kim YR. A disposable amperometric dual sensor for the detection of hemoglobin and glycated hemoglobin in a finger prick blood sample. Biosens Bioelectron. 2016; 85:331-336.

*Reason for exclusion*: Not a primary epidemiological study.

49. Krass I, He L, Fung D, Li J, Mirkov S, O'Brien J, et al. Pharmacy diabetes screening trial: protocol for a pragmatic cluster-randomised controlled trial to compare three screening methods for undiagnosed type 2 diabetes in Australian community pharmacy. BMJ Open. 2017;7(5): e016696.

*Reason for exclusion*: Not a primary epidemiological study.

50. Kenealy T, Garrett M, Harrison T, Sheridan J, Hatcher S. HbA1c screening in the community: lessons for safety and quality management of a point of care programme. BMJ Open Qual. 2019;8(3): e000533.

*Reason for exclusion*: Not a primary epidemiological study.

51. Nathan DM, Faghih M, Pirk L, Acker R. Accuracy of a point-of-care hemoglobin A1c assay. Clin Chem. 2019;65(5):634-641.

*Reason for exclusion*: Not a primary epidemiological study.

52. Brown D, Prather S. A diabetes screening and educational event in rural Alabama. J Am Pharm Assoc. 2020;60(2): e20-e23. doi: 10.1016/j.japh.2019.09.006.

*Reason for exclusion*: Not a primary epidemiological study.

53. Sivaprasad S, Narendran K, Shaik F, Vijayakumar B, Thangaraj K, Kumar K, et al. Protocol on a multicentre statistical and economic modelling study of risk-based stratified and personalised screening for diabetes and its complications in India (SMART India). BMJ Open. 2020;10(12): e042452.

*Reason for exclusion*: Not a primary epidemiological study.

54. Krass I, He L, Fung D, Mirkov S, O'Brien J, Li J, et al. Pharmacy diabetes screening trial (PDST): outcomes of a national clustered RCT comparing three screening methods for undiagnosed type 2 diabetes (T2DM) in community pharmacy. Diabet Med. 2023;40(7): e15000.

*Reason for exclusion*: Not a primary epidemiological study.

55. Younes N, Aoun M, Bou Assi N, el Eid R, Chebel M. Performance evaluation of a new fluorescent-based lateral flow immunoassay for quantification of hemoglobin A1c (HbA1c) in diabetic patients. Clin Chim Acta. 2023; 541:1-7.

*Reason for exclusion*: Not a primary epidemiological study. 
